# Spectrum of cryptococcal manifestations in a young male with human immunodeficiency virus

**DOI:** 10.1016/j.radcr.2024.09.008

**Published:** 2024-09-25

**Authors:** Lord Al-Sharrak, Alexander M. Satei, Rajbir S. Pannu, George M. Pappas

**Affiliations:** a Michigan State College of Osteopathic Medicine, East Lansing, MI, USA; b Department of Radiology, Trinity Health Oakland Hospital/Wayne State University School of Medicine, Pontiac, MI, USA; c Huron Valley Radiology, Ypsilanti, MI, USA

**Keywords:** Cryptococcus, Human immunodeficiency virus, AIDS, Brain lesion, CNS infection

## Abstract

We present the case of a young male with human immunodeficiency virus and a history of nonadherence to antiretroviral therapy who developed cryptococcal meningitis. This case highlights the diverse medical and clinical presentations of central nervous system cryptococcosis in an immunocompromised individual from a radiology perspective. CT and MR imaging demonstrated basal ganglia enhancement and leptomeningeal involvement, characteristic of this pathogen. This report underscores the significance of advanced imaging modalities, in particular MRI, in diagnosing cryptococcal meningitis. Additionally, other manifestations of cryptococcus, including within the thorax, are highlighted in the same patient. The combination of these findings, along with confirmatory cerebral spinal fluid analysis, are crucial to the rapid initiation of an appropriate antifungal regimen for treatment.

## Introduction

*Cryptococcus neoformans* is an encapsulated obligate aerobic opportunistic fungal organism that causes infections in immunocompromised patients, including those with human immunodeficiency virus (HIV), prior organ transplantation, diabetes, or hematological malignancy. This pathogen is composed of a capsular polysaccharide of glucuronoxylomannan. Cryptococcus infection occurs via inhalation of spores or yeast cells that deposit in the alveoli of the lungs [1]. The infectious cycle of the disease is a result of toxin production and a virulence factor expression. The main immune cell responsible for protection against cryptococcus involves generating Th1 and Th17 responses to recruit macrophages that effectively induce pathogen death, without excessive host tissue damage [2]. Diagnosing *C neoformans* in immunocompromised individuals presents a substantial diagnostic challenge due to subtle onset and nonspecific clinical manifestations. Progression of disease is rapid, causing neurological complications if not identified promptly. Advanced imaging is imperative to understanding the disease process, and detecting pathological features. Imaging modalities are often compared for sensitivity in detecting pathogenesis of disease. In this study, differential features of the disease are discussed through multiple imaging modalities to identify and manage disease progression and further reduce the burden of disease mortality.

## Case report

A 27-year-old male with a personal history of HIV, diagnosed 4 years prior to presentation and noncompliant with highly active antiretroviral therapy (HAART), became unresponsive shortly after arriving at our emergency room. Physical examination demonstrated dilated pupils, rigidity of the upper and lower extremities, inability to follow commands, urinary incontinence, and skin findings suggestive of prior intravenous drug abuse. Lungs were clear to auscultation and a regular heart rate and rhythm were appreciated.

Laboratory investigations demonstrated leukocytosis of 14.0 K/mcL and elevated lactate of 5.1 mmol/L. Urine drug screen and urinalysis were negative. Absolute CD4 count was 25 cells/mm^3^ and CD4 percentage was 6%. Peripheral blood cultures were positive for *C neoformans*.

**Fig. 1:**
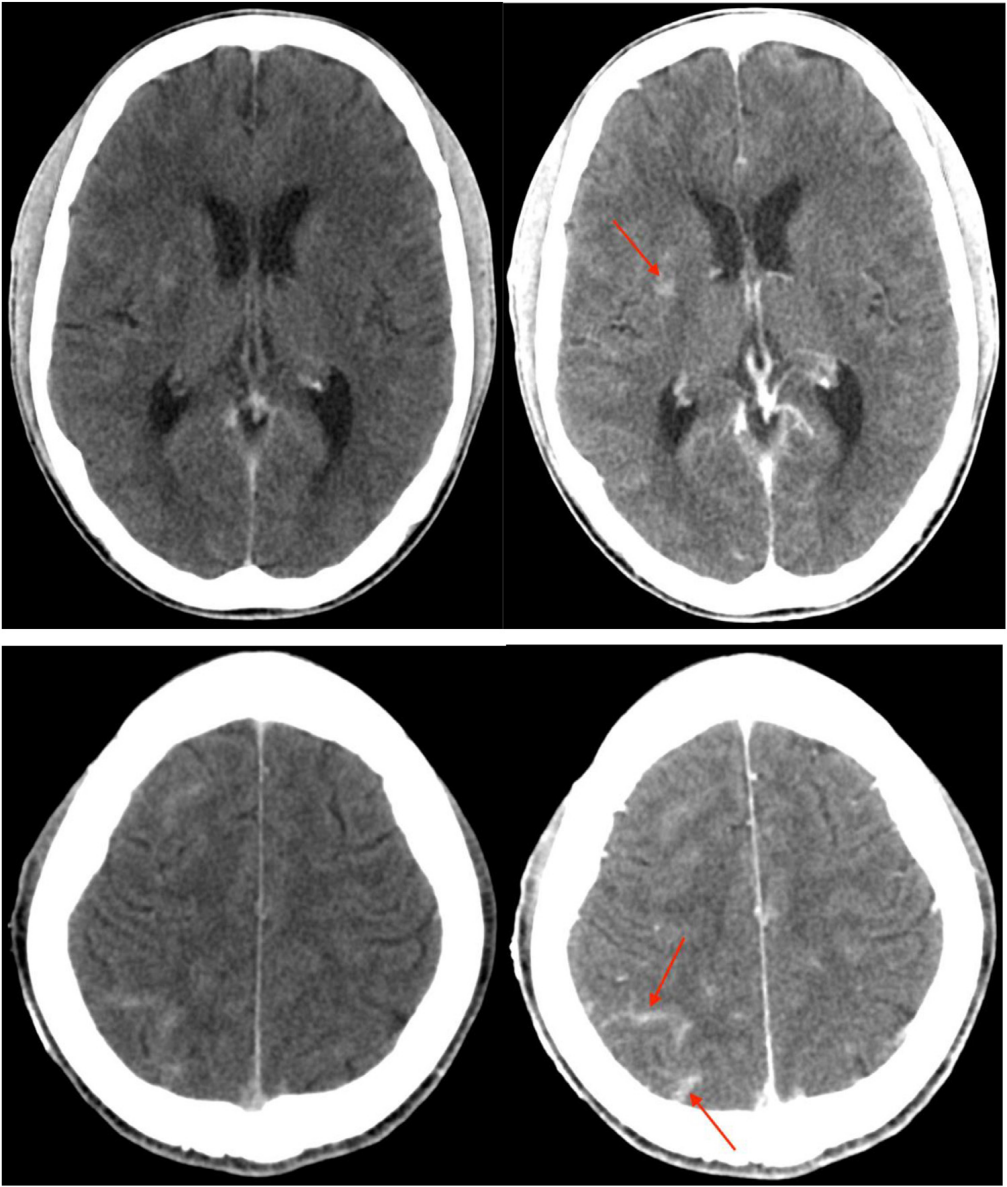
Axial pre (A) and postcontrast (B) CT images demonstrate small regions of enhancement within the basal ganglia, more conspicuous on the right (red arrow). On a lower slice of the brain, axial pre (C) and postcontrast (D) images demonstrate sulcal hyperdensity, most pronounced within the right parietal lobe (red arrows), and likely relating to leptomeningeal enhancement.

**Fig. 2:**
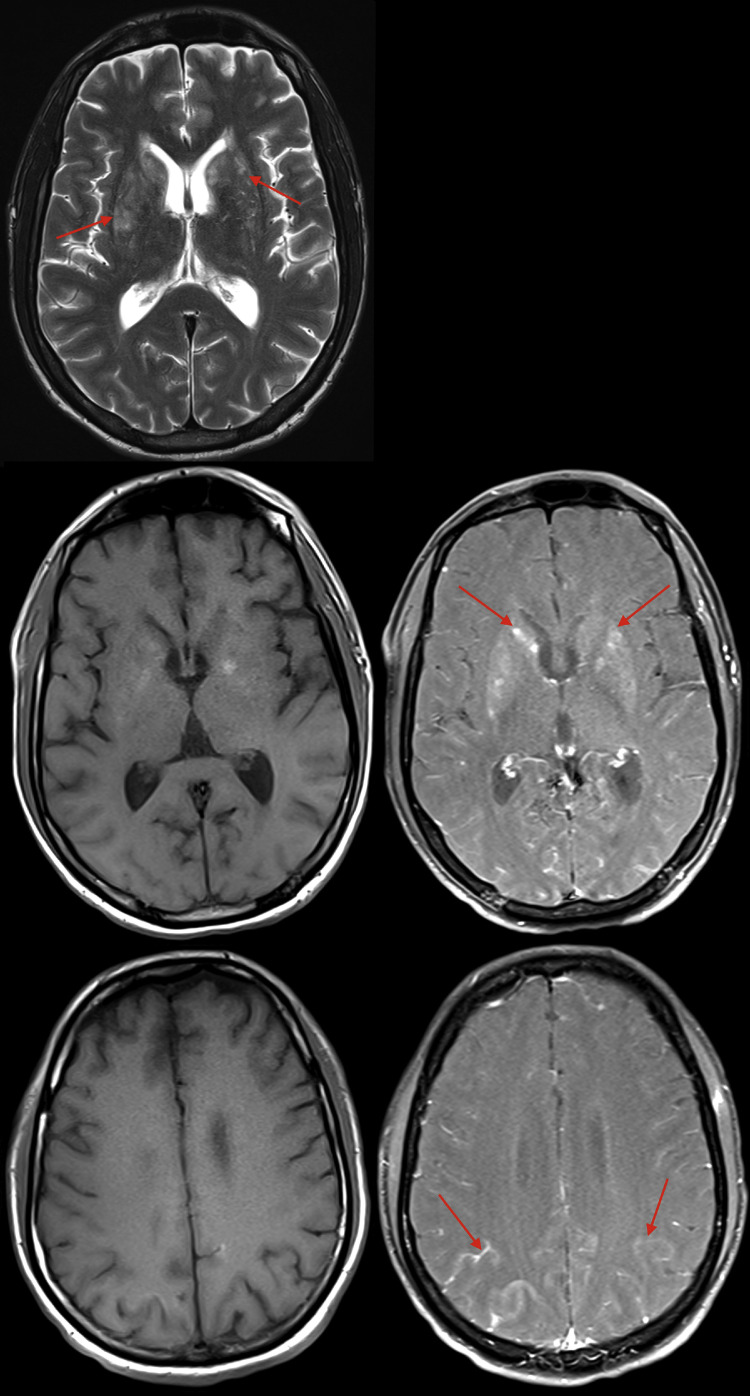
Axial T2-weighted MRI sequence (A) demonstrates hyperintensities involving the bilateral corpus striatum (red arrows). Axial T1-weighted sequences pre- (B) and postcontrast (C) demonstrate multifocal nodular enhancement involving the bilateral corpus striatum (red arrows); these lesions demonstrate intrinsic T1 hyperintensity. Additionally, axial T1-weighted sequences pre (D) and postcontrast (E) demonstrate leptomeningeal enhancement involving the bilateral frontal and parietal lobes.

The patient was started on normal saline, and an anti-seizure regimen of oxcarbazepine 150 mg twice daily. Computed tomography (CT) of the head with and without contrast was obtained for evaluation of intracranial involvement of the infection. Enhancing lesions within the basal ganglia (Figs. 1A and B) as well leptomeningeal enhancement within the parietal lobe (Figs. 1C and D) were noted. Follow-up magnetic resonance imaging (MRI) of the brain with and without contrast was performed the subsequent day. This revealed T2 hyperintensities within the bilateral corpus striatum (Fig. 2A), with nodular multifocal enhancement within this region (Figs. 2B and C). Additionally, leptomeningeal enhancement was redemonstrated (Figs. 2D and E). Cerebrospinal fluid (CSF) analysis was positive for cryptococcal antigen, demonstrated high opening pressure, and subsequent cultures grew 2+ *Cryptococcus neoformans*.

**Fig. 3:**
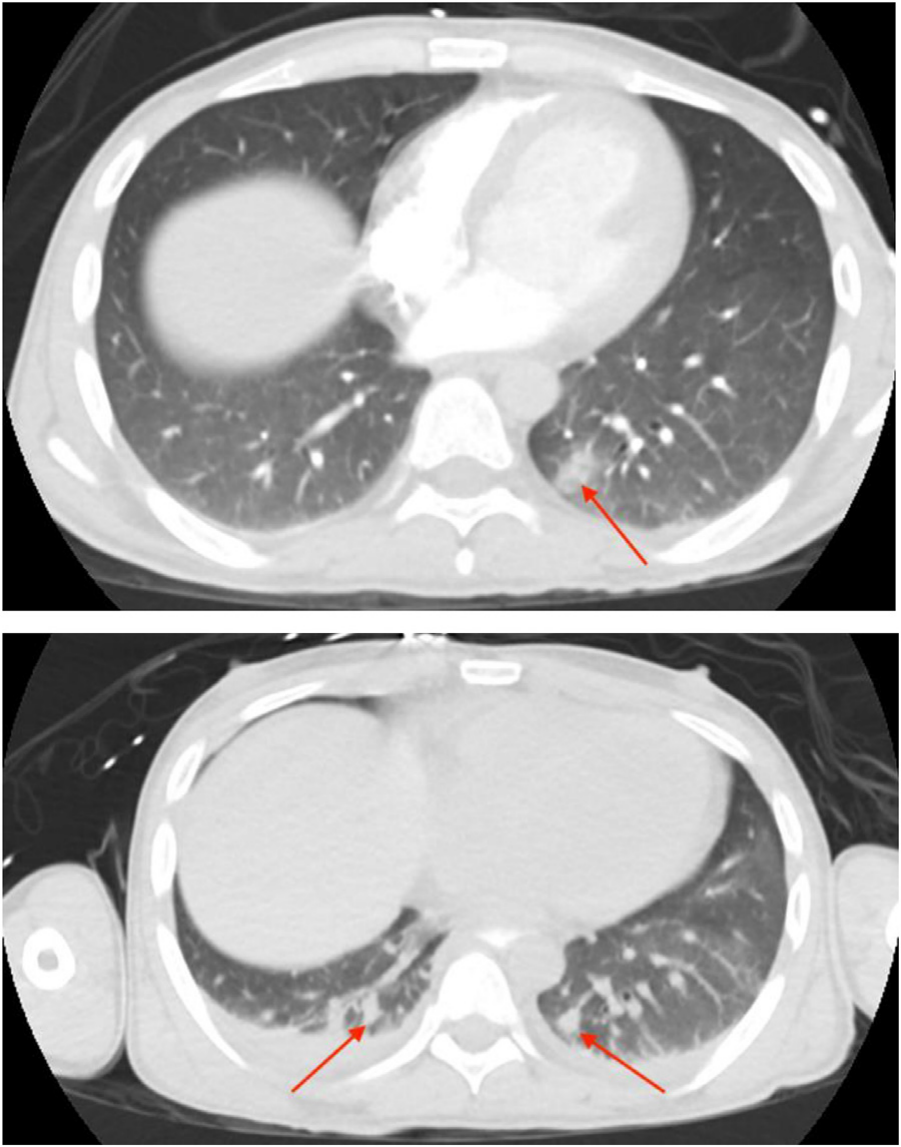
CT of the chest with contrast near the time of admission (A) and a month later (B) demonstrates patchy opacities within the left lower lobe which become more nodular and bilateral distribution (red arrows) on the subsequent examination consistent with the patient's multifocal pneumonia.

Given the confirmation of cryptococcal meningitis, the patient was admitted to the intensive care unit and started on liposomal amphotericin B and flucytosine. Chest radiography demonstrated mild diffuse interstitial prominence, and therefore a CT of the chest was obtained to evaluate for intrathoracic disease. CT of the chest demonstrated opacities within the left lower lobe concerning for cryptococcal left lower lobe pneumonia (Fig. 3A); on a follow-up CT obtained a month later, findings appeared to have worsened and involved the basilar lungs bilaterally (Fig. 3B).

The patient remained in the hospital for nearly a month, during which time subsequent CSF analyses were negative for Cryptococcus. Despite the worsening findings on chest CT, the patient refused further inpatient care or in-patient rehabilitation. Ultimately, he was discharged with home care and counseled on HAART adherence.

## Discussion

The acquired immune deficiency syndrome (AIDS) epidemic resulted in a shift in the incidence of cryptococcosis, increasing from 1 case per million people annually to 5%-10% of AIDS patients in the USA [3]. Cryptococcus infections account for 15% of AIDS-related deaths worldwide. *C. neoformans* is considered the third most common pathogen causing CNS infection in AIDS, and approximately 5% of AIDS patients with CD4 counts less than 200 cells/mm^3^ will develop CNS findings related to cryptococcosis.

Cryptococcal burden on global health accounts has been shown to pose a threat predominantly to immunocompromised individuals due to the emergence of antifungal-resistant variants. The role of diagnostic techniques, especially imaging, has therefore been increasingly important to reduce the threat of *C. neoformans*. Typically, the first organ involved is the lungs, in the form of cryptogenic organizing pneumonia. This is followed by the central nervous system as cryptococcal meningitis and then the bloodstream as cyrptococcemia.

Symptoms of cryptococcal meningitis include fever, headache, and vomiting. Less common symptoms include altered mental status, confusion, drowsiness, fatigue, weight loss, and weakness. The diagnosis of cryptococcal meningitis is established through India ink microscopy, CSF cultures, and cryptococcal antigens in CSF; CSF fungal culture is the gold standard for diagnosis. The World Health Organization guidelines in 2022 recommended that all HIV patients with CD4 counts less than 200 cells/µl be screened for *C neoformans* [4].

Imaging findings are crucial to the diagnosis of CNS cryptococcosis. On nonenhanced CT scans, findings can be nonspecific, including atrophy, hydrocephalus, and edema from mass lesions [5]. On MRI, cryptococcomas, which are parenchymal lesions of encapsulated cryptococcus, can be visualized. Cryptococcomas demonstrate restriction on diffusion weighted imaging (DWI), with corresponding hypointensity on apparent diffusion coefficient maps (ADC), and hyperintensity on T2/fluid attenuated inversion recovery (FLAIR) sequences. Small lacunar-type infarcts are associated with CNS cryptococcosis, best appreciated on DWI/ADC sequences. Contrast-enhanced T1 sequences demonstrate pachymeningeal and leptomeningeal enhancement, with variable enhancement of cryptococcomas. Additionally, dilated perivascular spaces resulting in pseudocysts may develop. Negative brain imaging was seen in 47% of CT scans and 8% of MR scans; therefore, MRI provides superior specificity for the diagnosis of CNS cryptococcus [6].

In the management of cryptococcal meningitis in an HIV infected patient, the timing of HAART is important. According to the ACTG5164 trial, the initiation of HAART in HIV positive cryptococcal meningitis patients within 14 days of diagnosis correlated with reduced mortality and AIDS progression, compared with initiating therapy within 4 weeks. Thus, timely management of symptoms and imaging is important to reduce disease burden [7]. Treatment of the pathogen itself also involves intravenous antifungals, typically fluconazole or amphotericin B.

## Learning points

• MRI is superior to CT imaging in the accurate diagnosis and assessment of cerebral cryptococcal meningitis, particularly in HIV-positive patients, yet negative findings may not rule out disease. Correlation with CSF analysis is recommended in clinically suspected cases.
• Prompt recognition of imaging findings, such as basal ganglia enhancement and leptomeningeal involvement, is crucial in the prompt diagnosis of CNS cryptococcosis.
• Consistent and timely HAART administration can prevent and help treat opportunistic infections like *C neoformans* in HIV-positive patients.

## Patient consent

Informed consent was obtained from the patient for publication of this report and accompanying images.
